# A Hybrid Secure Scheme for Wireless Sensor Networks against Timing Attacks Using Continuous-Time Markov Chain and Queueing Model

**DOI:** 10.3390/s16101606

**Published:** 2016-09-28

**Authors:** Tianhui Meng, Xiaofan Li, Sha Zhang, Yubin Zhao

**Affiliations:** 1 Department of Mathematics and Computer Science, Freie Universität Berlin, Berlin 14195, Germany; tianhui.meng@fu-berlin.de; 2 Shenzhen Institute of Radio Testing & Tech., Shenzhen 518000, China; lixiaofan@srtc.org.cn; 3 Shenzhen Institutes of Advanced Technology, Chinese Academy of Sciences, Shenzhen 518055, China; zhaoyb@siat.ac.cn

**Keywords:** Markov chain, queueing model, side-channel attacks, random padding

## Abstract

Wireless sensor networks (WSNs) have recently gained popularity for a wide spectrum of applications. Monitoring tasks can be performed in various environments. This may be beneficial in many scenarios, but it certainly exhibits new challenges in terms of security due to increased data transmission over the wireless channel with potentially unknown threats. Among possible security issues are timing attacks, which are not prevented by traditional cryptographic security. Moreover, the limited energy and memory resources prohibit the use of complex security mechanisms in such systems. Therefore, balancing between security and the associated energy consumption becomes a crucial challenge. This paper proposes a secure scheme for WSNs while maintaining the requirement of the security-performance tradeoff. In order to proceed to a quantitative treatment of this problem, a hybrid continuous-time Markov chain (CTMC) and queueing model are put forward, and the tradeoff analysis of the security and performance attributes is carried out. By extending and transforming this model, the mean time to security attributes failure is evaluated. Through tradeoff analysis, we show that our scheme can enhance the security of WSNs, and the optimal rekeying rate of the performance and security tradeoff can be obtained.

## 1. Introduction

Wireless sensor networks (WSNs), which are enabled by the developments of wireless communications, micro-electro-mechanical systems (MEMS) technology and digital electronics, have found their way into a wide variety of applications and systems with vastly varying requirements and characteristics. In accordance with the vision of [1], WSNs are slowly becoming an integral part of our daily lives. The sensing technology combined with processing power and wireless communication makes it lucrative for being exploited in abundance in the future [2]. Recently, considerable amounts of research efforts have enabled the actual implementation and deployment of sensor networks tailored to the unique requirements of certain sensing and monitoring applications [3,4,5].

Since these networks are usually deployed in remote areas and left unattended, they should be defended by security mechanisms against attacks, such as physical tampering, node capture, eavesdropping, denial of service, etc. Unfortunately, traditional security mechanisms with high overhead are not feasible for the resource-constrained sensor nodes [6]. An important issue of WSNs is how to preserve sensitive measurements taken from everyday life where data privacy is an essential aspect. In many scenarios, the confidentiality of propagated data can be considered critical. For instance, in a healthcare system, data from sensors might measure patients’ health information, such as heartbeat and blood pressure details. Data privacy can be simply defined as a process in which private data can be overheard and decrypted by adversaries or other trusted participating sensor nodes [7], but it can still provide a mechanism that prevents them from recovering sensitive information, i.e., control disclosure of any information about the data.

Securing cryptographic implementations in WSNs against side-channel attacks is one of the most important challenges [8], which is not covered by traditional notions of cryptographic security [9]. Among side-channel attacks, timing attacks, whose remote feasibility has been proven by Brumley and Boneh [10,11], make a practical threat against sensor network systems. Energy is an essential resource of WSNs, so the implementations of cryptographic algorithms in such systems often perform computations in non-constant time due to performance optimization. The timing attack involves algorithms that have non-constant execution time, and this can potentially leak secret information. As represented in [12], the operating system running on the sensor nodes is event-driven and extremely optimized in terms of memory consumption, which suggests that the timing side-channel is practical in WSNs.

In this paper, we propose a secure scheme to defend WSNs from timing attacks. The rekeying process with an optimal rate is introduced into the WSNs, and random paddings are interposed in the processing time to mitigate the information leakage of the timing side-channel. Our contributions are now summarized as follows:

- In order to proceed to a quantitative treatment of the performance-security tradeoff of WSNs, we propose a hybrid continuous-time Markov chain (CTMC) and queueing model for the system under the specific threat of timing attacks.
- We have shown the measures’ formulation, including both security and performance attributes, and the optimal tradeoff between the two.
- Experimental evaluations demonstrate the effectiveness of the random padding countermeasure against timing attacks and the tradeoff improvement one can obtain from the proposed scheme.

The remainder of this paper is structured as follows. In Section 2, we overview the current state-of-art related works. Section 3 shows the considered multi-gateway clustered topology and rekeying management method as the system architecture. A hybrid model that employs the hybrid CTMC and queueing model for a WSN under timing attacks is proposed in Section 4. In this section, we describe the state-transition model and the scheduling method. The system metrics on which the evaluation is based are addressed in Section 5. Section 6 gives the theoretical model analysis of the security and performance attributes using quantitative assessment methods. Experimental evaluation of the general WSN system is presented in Section 7. Finally, the paper is concluded in Section 8.

## 2. Related Work

A WSN is a collection of nodes organized into a cooperative network [13]. Applications of WSNs are growing rapidly, which range from indoor deployment scenarios in offices to outdoor deployments in adversarial territory. However, due to their deployment environments and resource limitations, these networks are vulnerable to many security threats that can deteriorate the performance [14].

These attacks are often classified as external attacks and internal attacks [15]. In an external attack, the attacker node is not an authorized participant of the sensor network. Adversaries can severely limit the usefulness of a WSN through denial-of-service (DoS) attacks [16,17]. An attacker may simply disrupt the network operation by broadcasting a high-energy signal. If the transmission is powerful enough, the entire system communication could be jammed. The standard defense against jamming involves various forms of spread-spectrum or frequency hopping communication [18]. However, cryptographically-secure spread-spectrum radios are not commercially available. In addition, this defense is not sufficiently secure against node capture attacks and cryptographic key extracting [14].

Node compromise is the major problem in sensor networks that leads to internal attacks. In contrast to disabled nodes, compromised nodes actively seek to disrupt or paralyze the network [19]. Routing and data forwarding are essential services for enabling communication in sensor networks. Unfortunately, many routing protocols suffer from security vulnerabilities [20]. The simplest attacks involve injecting malicious routing information into the network, resulting in routing inconsistencies.

Another area that has received a great deal of attention in WSN security is key management. A taxonomy of key management protocols in WSNs is described in [21].

Non-constant execution time can be caused by conditional branching and various optimization techniques. If such operations involve secret parameters, these timing variations can leak some information, and a careful statistical analysis could even lead to the total recovery of these secret keys. Because timing attacks gain secret information from the response times and rather than brute force attacks or theoretical weaknesses in the algorithms, they are a real threat to WSNs as an internal attack. However, this threat is not covered by traditional notions of cryptographic security [8]. It was commonly believed that timing attacks can be directed only towards smart cards or affect the inter-process locally, but more recent research reveals that remote timing attacks are also possible and should be taken into consideration [10,11].

The interposition of random delays in the cryptographic algorithm execution flow is a simple, but rather effective countermeasure against side-channel and fault attacks by mitigating the information leakage. Random delays are easily deployed even if the source code of the application is not at hand. They are widely used for the protection of cryptographic implementations in embedded devices [22]. To date, based on random delay insertion, a processor architecture resistant to side-channel attacks is proposed in [23] using a combination of randomized scheduling, randomized instruction insertion and randomized pipeline-delay. The researchers in [24] present a design and hardware implementation of asynchronous AES with random noise injection for improved side-channel attack resistance.

## 3. Regarded System Architecture

**Figure 1:**
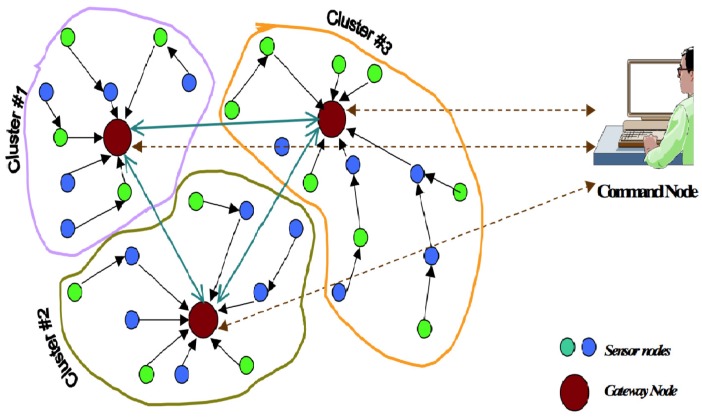
Architecture of a multi-gateway clustered sensor network.

We adopt the sensor network model in Figure 1, which is proposed by Younis et al. [25]. In this model, the network consists of a large number of sensor nodes distributed over an area of interest. There is at least one command node in charge of the system. The model also introduces some super-nodes, called gateway nodes, in addition to the sensor nodes. The gateway nodes have more computational, energy, memory and communication resources. They act as a gateway between sensor nodes and the end user, as they typically forward data from the WSN on to a command node [26]. As depicted in Figure 1, the gateways partition the sensors into different distinct clusters. Each cluster is composed of a gateway node and a set of sensor nodes. The operations within a cluster are independent of each other. The cluster sensor nodes transmit the gathered and integrated data to the gateway node of their cluster.

It is assumed that the gateway nodes are deployed in safe places and cannot be captured by the adversary; while the other nodes in the cluster may be captured and compromised. For the key management, we adopt the scheme proposed by Eschenauer and Gligor [27]. The management scheme triggers the rekeying phase regularly to handle the timing attacks from inside or outside attackers, and our goal is to indicate the optimal rekeying rate. In our approach, the sensor nodes use a symmetric key mechanism. A cluster key is used for the encryption and decryption operations of all data between the sensors and the gateway node. Before being deployed, the cluster key is programmed into the memory of the sensors. Sensor and gateway keys are stored in the flash RAM and, hence, can be renewed in the rekeying phase. We assume that the time to perform a rekeying operation is measured based on a generic Diffie–Hellman protocol (GDH) [28,29]. At the same time, random delays are padded for each gateway node response message to mitigate the information leakage of the timing side-channel.

## 4. The System Model

**Figure 2:**
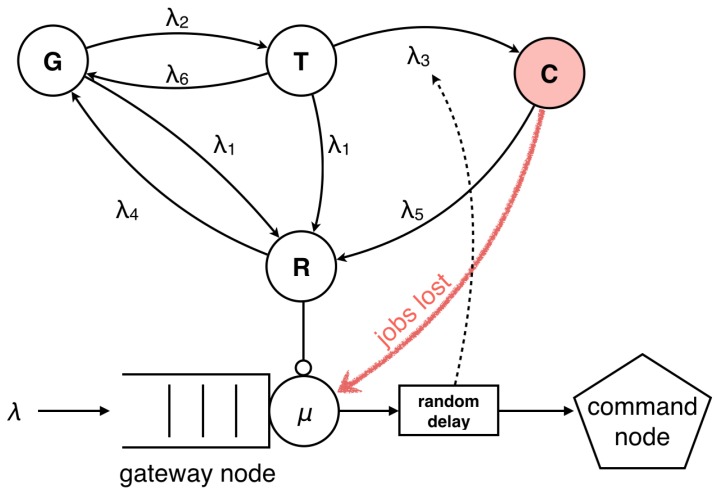
State transition diagram for a WSN under timing attacks.

In order to analyze the performance and security attributes of WSNs under the threat of timing attacks quantitatively, we need to combine the behaviors of an attacker who is trying to gain the sensitive information in conjunction with the countermeasures taken by the system. Therefore, we developed a hybrid CTMC and queueing model, shown in Figure 2, that takes into account the behavior of both sides. The goal is to identify optimal system settings to maximize the security and performance tradeoff metric described in Section 5.3.

The state transition model represents the system behavior under a specific attack and given system configuration that depends on the actual security requirements; whereas, the queueing model is proposed to exhibit the data transmission between sensors and gateway nodes. A coming job joins a queue before it is processed and forwarded to a command node. The states and parameters of the CTMC state transition model are summarized here:

- *G* Good state in which the WSN works properly
- *T* Timing attack state
- *C* Compromised state after the attacker knows the secret of the system
- *R* Rekeying state in which the system renews its cluster key
- $\lambda_{1}$ rate at which the system launches the rekeying process in state *G* and state *T*
- $\lambda_{2}$ rate at which an attacker triggers a timing attack on the system
- $\lambda_{3}$ rate at which a timing attack succeeds to break the cluster key
- $\lambda_{4}$ rate at which the WSN is brought back to the good state by the rekeying process
- $\lambda_{5}$ rate at which the system launches the rekeying process in the compromised state *C*
- $\lambda_{6}$ rate at which the attacker successfully breaks the key, while failing to access legitimately-authorized information.

### 4.1. Attack Model

The WSN is vulnerable to timing attacks in which the attacker in the worst case will eventually obtain the secret cluster key. In order to analyze the security properties of the proposed scheme, it is necessary to consider the actions undertaken by an adversary, as well as the system’s response to the adversary.

In timing attacks on a WSN, an attacker first compromises a sensor node or mimics a sensor node to send data to the gateway node. In addition, the attacker records each response time for a certain query and tries to find clues to the cluster key by comparing differences in the response times. On the condition that the attacker breaks the cluster key from the timing results, he or she can attack the secrecy and authenticity of communication channels. Obviously, this requires the attacker to spend effort, where we use time to represent the attacking effort. This time is modeled as a random variable, which may follow one distribution function depending on the nature of the attack. Deterministic, exponential, hypo-exponential, hyper-exponential, gamma, Weibull and log-logistic, etc., are some of the distribution functions that make sense in the context of security analysis [30]; while we use an exponential distribution to model the attacker arrival time and the time a timing attack takes.

### 4.2. Security and Performance Model

In the presence of inside and outside attacks, our secure scheme for a WSN is concerned primarily with the evaluation of the three attributes: confidentiality (sensitive information is not disclosed to unauthorized member), integrity (data and programs are modified only in a authorized manner) and availability (readiness for usage).

The upper part of Figure 2 shows a CTMC model representing the states of the system. After initialization, the system starts to operate properly in the good state *G*. The system is under the specific threat of timing attacks conducted by random attackers. We describe the events that trigger transitions among states in terms of transition rates. It is assumed that there is only one attacker in the system at a time. If an attack happens, the system is brought to the timing attack state *T* at rate $\lambda_{2}$. In this state, the attacker tries to break the cluster key by taking time observations. Therefore, while the system is in state *T*, the attacker is not yet able to access confidential information.

It takes a certain time to perform the timing attack after which the attacker may know the cluster key, and the system moves to the compromised state *C* at rate $\lambda_{3}$. Consequently, $\lambda_{3}^{-1}$ is the mean time a timing attack takes. There is a possibility indicated by the arc $\lambda_{6}$ that the attacker successfully breaks the key, while he or she fails at accessing the data or just fails to conduct a successful timing attack. If the attacker succeeds in determining the cluster key through time measurements, confidential data will be disclosed, which is assumed to incur a high cost. This can only happen if the system is in the compromised state *C*, and we call the incident of entering the compromised state a security failure. In this state, all of the data transited between sensors and gateway nodes are not secure any more; therefore, they must be repeated and do not contribute to the throughput. The lost jobs are represented by the red arc in Figure 2.

Renewing the cluster encryption key can prevent or interrupt a timing attack. The arcs from other states to state *R* represent these operations. The rekeying rate is the parameter one can tune at the command node. It indicates how often the system launches the rekeying process. The rate $\lambda_{1}$ is the rekeying rate when the system is in the good state *G* or in the timing attack state *T*. The considered WSN has intrusion detection mechanisms running that can find indicators of compromised behavior, in which case the system will trigger the rekeying process more frequently. The intrusion detection mechanism does not trigger the rekeying immediately because of the rekeying cost to the system. Therefore, in the compromised state *C*, we assume that the rekeying process is triggered at a different rate, $\lambda_{5}=n\lambda_{1}$. The parameter *n* is called the coefficient of rekeying in the compromised state because it represent the relationship between the rekeying rate in the good state and the rekeying rate in the compromised state. All of these three paths transfer the WSN cluster to the rekeying state *R* from which it will finally return to the initial state *G*. The challenge is to find an optimal value for the rekeying interval. The rekeying should in the optimal case happen before or soon after the system enters the compromised state.

In the rekeying state, the sensors do not transit any data to the gateway, and some jobs will be lost in this state. As a result, the system throughput is degraded. The rekeying process will bring the system back to the initial state *G* at rate $\lambda_{4}$. The mean time to perform the rekeying process is $\lambda_{4}^{-1}$.

The lower part of Figure 2 shows the queueing model proposed to exhibit the performance attribute of the WSN. The data collected and preprocessed by sensors are transmitted to the gateway node before forwarded to a command node, which is represented by a queue. The parameter *λ* indicates the arrival rate, and *μ* presents the service rate of the gateway node, including the data transmission time. When random delays are interposed in the gateway node, the attacker needs more samples to average and successfully guess the cluster key. Therefore, it takes more time for him or her to conduct the timing attack. As a result, the attacking rate $\lambda_{3}$ decreases as this mitigation method is taken, which is represented by the dashed arc in the figure.

## 5. Metrics

After defining the model and its parameters, we establish the measures we want to investigate in this section. We present security and performance metrics, respectively, which are illustrated in Table 1. Since the sensor nodes carry limited battery resources, WSN applications and communication protocols focus primarily on power conservation. As a result, communication is an important factor, since data transmission mainly influences energy consumption.

### 5.1. Security Metrics

As security measures, we define system cost and confidentiality, which are functions of the steady-state probabilities of the CTMC model. The steady-state probabilities $\pi_{i}$ may be interpreted as the proportion of time that the CTMC spends in state *i*, where i∈{R,G,T,C}. The WSN suffers from cost in two states, the compromised state *C* and the rekeying state *R*. The system loses sensitive information in the compromised state, and cost is also incurred when the clustered sensor network deploys a rekeying process due to the extra communication. The rekeying cost and the data disclosure cost are both interpreted as the proportion of system life time, that is the steady-state probability of the CTMC. We define a weight *w* and its complement 1−w for the two kinds of cost. We use normalized weights for simplicity. Mathematically, the system cost is computed with:

(1) $$\begin{array}{ccccccccc} \Theta & & = & & C_{rekeying} & & + & & C_{disclosure}\\ & & = & & w\pi_{R} & & + & & \left(1-w\right)\pi_{C}\; \end{array}$$

where $\pi_{i},i\in \left\{R,C\right\}$ denotes the steady-state probability that the continuous-time Markov process is in state *i*. 0≤w≤1 is the weighting parameter used to share the relative importance between the loss of sensitive information and the power consumption needed to rekey regularly.

If a timing attack on the WSN cluster is successful, the attacker obtains the cluster key and can browse unauthorized data thereafter. The entered states denote the loss of confidentiality. Therefore, the steady-state confidentiality measure can be computed as:

(2) $$\Lambda =1-\pi_{C}\;.$$

For quantifying the reliability of a software system, mean time to failure (MTTF) is a widely-used reliability measure. We also use the mean time to security failure (MTTS) metric as a measure for quantifying the security of WSNs in Section 6.3.

### 5.2. Performance Metrics

The performance metrics of interest describe the system in terms of throughput, completion time or response time, as defined in queueing theory or networking. Here, we use the throughput as the performance metric for the WSN. By Little’s law, the throughput (denoted by *X*) is defined as:

(3) $$X=\frac{E[N]}{E[R]}\;.$$

For a queue, the throughput equals the average number of jobs in the queueing station (E[N]) divided by the average time a job spends in the queueing station (E[R]).

### 5.3. Tradeoff Metric

In order to investigate how the security of WSNs will interact with performance, we define a tradeoff metric. An objective function formed from the product of the security attribute confidentiality and system throughput is created to demonstrate the tradeoff situation:

(4) T=Λ×X.

As a WSN designer, one may look forward to maintaining the confidentiality of sensitive information with higher throughput, as for the tradeoff measure, the larger the better. In the next section, we will evaluate these measures by computing the steady-state probability of the CTMC and solving the queueing model.

## 6. Model Analysis

In this section, we derive and evaluate the security and performance attributes using methods for the quantitative assessment of dependability, known as the dependability attributes, e.g., reliability, availability and safety, which have been well established quantitatively.

### 6.1. CTMC Steady-State Probability Computation

For the system security attributes, we have described the system’s dynamic behavior by a CTMC model with the state space $X_{s}=\left\{R,G,T,C\right\}$ and the transitions between these states. In order to carry out the security quantification analysis, we need to determine the stationary distribution of the CTMC model.

The steady-state probabilities $\left\{\pi_{i},i\in X_{s}\right\}$ of the CTMC can be computed by solving the system of linear equations [31]:

(5) πQ=0,

where $\pi =\left[\pi_{R},\pi_{G},\pi_{T},\pi_{C}\right]$ and Q is the infinitesimal generator (or transition-rate matrix), which can be written as:

(6) $$Q=\begin{array}{cccccc} & R & G & T & C\\ \begin{array}{c} R\\ G\\ T\\ C \end{array}( & \begin{array}{c} -\lambda_{4}\\ \lambda_{1}\\ \lambda_{1}\\ \lambda_{5} \end{array}\; & \begin{array}{c} \lambda_{4}\\ -\lambda_{1}-\lambda_{2}\\ \lambda_{6}\\ 0 \end{array} & \begin{array}{c} 0\\ \lambda_{2}\\ -\lambda_{1}-\lambda_{3}-\lambda_{6}\\ 0 \end{array} & \begin{array}{c} 0\\ 0\\ \lambda_{3}\\ -\lambda_{5} \end{array} & ) \end{array}$$

In addition, we have the total probability relationship:

(7) $$\sum_{i}\pi_{i}=1\;i\in X_{s}.$$

The transition-rate matrix Q describes the dynamic behavior of the security model, as shown in Figure 2. The first step towards quantitatively evaluating security attributes is to find the steady-state probability vector *π* of the CTMC states by solving Equations (5) and (7). We obtain:

(8) $$\begin{array}{cc} \pi_{R}= & \;\frac{\left[\left(\lambda_{1}+\lambda_{2}\right)\left(\lambda_{1}+\lambda_{3}\right)+\lambda_{1}\lambda_{6}\right]\lambda_{5}}{\phi },\\ \pi_{G}= & \;\frac{\left(\lambda_{1}+\lambda_{3}+\lambda_{6}\right)\lambda_{4}\lambda_{5}}{\phi },\\ \pi_{T}= & \;\frac{\lambda_{2}\lambda_{4}\lambda_{5}}{\phi },\\ \pi_{C}= & \;\frac{\lambda_{2}\lambda_{3}\lambda_{4}}{\phi }\;, \end{array}$$

for the sake of brevity, where $\phi =\left(\lambda_{1}+\lambda_{4}\right)\left(\lambda_{1}+\lambda_{3}+\lambda_{6}\right)\lambda_{5}+\left[\left(\lambda_{1}+\lambda_{4}\right)\lambda_{5}+\left(\lambda_{4}+\lambda_{5}\right)\lambda_{3}\right]\lambda_{2}$.

Given the steady-state probabilities of CTMC model, the confidentiality measure *Λ* and *Θ* measures can be computed via Equations (1) and (2):

(9) $$\Theta =w\frac{\left[\left(\lambda_{1}+\lambda_{2}\right)\left(\lambda_{1}+\lambda_{3}\right)+\lambda_{1}\lambda_{6}\right]\lambda_{5}}{\phi }+\left(1-w\right)\frac{\lambda_{2}\lambda_{3}\lambda_{4}}{\phi }\;,$$

(10) $$\Lambda =1-\frac{\lambda_{2}\lambda_{3}\lambda_{4}}{\phi }\;.$$

### 6.2. Throughput Analysis

Let the total system life time be *T*. The steady-state probabilities $\pi_{i}$ can be interpreted as the proportion of time that the CTMC spends in state *i*. In the good state *G* and timing attack state *T*, the number of jobs precessed is $\lambda (\pi_{G}+\pi_{T})T$, given that the queues are stable (λ<μ). While in the rekeying state *R*, the sensors do not transmit any data to the gateway node. In the compromised state *C*, all of the data dispatched to the gateway node are not secure, so they do not contribute to the throughput. Therefore, the system throughput is:

(11) $$\begin{array}{cc} X= & \frac{\lambda (\pi_{G}+\pi_{T})T}{T}\\ = & (\pi_{G}+\pi_{T})\lambda \;. \end{array}$$

### 6.3. CTMC with Absorbing State: MTTSF Analysis

**Figure 3:**
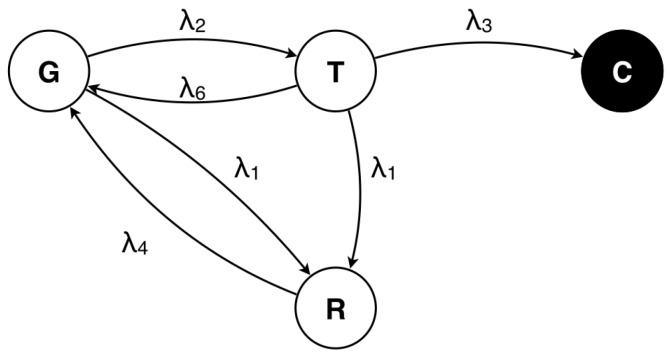
CTMC model with an absorbing state.

Mean time to failure (MTTF) provides the mean time it takes for the system to reach one of the designated failure states, given that the system starts in a good state. We use the mean time to security failure (MTTSF) as a measure for quantifying the security of the WSN. MTTF or MTTSF can be evaluated by making the compromised state of the CTMC an absorbing state, as shown in Figure 3. Once the system reaches the absorbing state (the compromised state *C*), the probability of moving out of this state is zero, i.e., there are no outgoing arcs from such states.

Given a CTMC with one absorbing state, we may enter this chain at some state *i* with probability $\alpha_{i}$. For each state that is visited, the time before going to the next state follows an exponential distribution. Thus, the time required to reach the absorbing state from an initial state *i* is a sum of samples from exponential distributions. The mean time to absorption can be solved easily by introducing the phase-type distribution (PH distribution). For a given CTMC, a PH distribution is defined as the distribution of the time to absorption that can be observed along the paths in a CTMC with one absorbing state [32]. The first moment of a PH distribution exactly expresses the mean time to absorption in an absorbing CTMC:

(12) $$E\left[X\right]=-\underline{\alpha }T^{-1}\underline{1}\;,$$

where $\underline{\alpha }=\left\{\alpha_{i},i\in X_{s}\right\}$ is the row vector of initial probabilities of the states and $\underline{1}$ is the column vector of ones. Substituted into our model parameters, we get:

(13) $$MTTSF=\frac{\left(\lambda_{1}+\lambda_{4}\right)\left(\lambda_{1}+\lambda_{2}+\lambda_{3}+\lambda_{6}\right)}{\lambda_{2}\lambda_{3}\lambda_{4}}\;.$$

When a software lifetime is given as $T_{l}$, we assume the supremum to be denoted by $sup(T_{l})$. In order to ensure system security, the MTTSF should be larger than the $sup(T_{l})$. That is:

(14) $$\frac{\left(\lambda_{1}+\lambda_{4}\right)\left(\lambda_{1}+\lambda_{2}+\lambda_{3}+\lambda_{6}\right)}{\lambda_{2}\lambda_{3}\lambda_{4}}>sup\left(T_{l}\right)\;.$$

Given the system parameters, the required rekeying rate $\lambda_{1}$ can be obtained by solving the inequality Equation (14). It has two roots, and we only take the positive root as:

(15) $$\begin{array}{c} \lambda_{1}>\frac{1}{2}\left(-\lambda_{2}-\lambda_{3}-\lambda_{4}-\lambda_{6}+\sqrt{\sigma^{2}}\right)\;, \end{array}$$

in which:

(16) $$\begin{array}{cc} \sigma^{2}= & \;\lambda_{2}^{2}+{\left(\lambda_{3}-\lambda_{4}+\lambda_{6}\right)}^{2}\\ & +2\lambda_{2}\left(\lambda_{3}-\lambda_{4}+\lambda_{6}+2\lambda_{3}\lambda_{4}sup\left(T_{l}\right)\right). \end{array}$$

Then, the system rekeying rate can be determined for a certain software lifetime. After determining the security and performance measures for the model, we conduct the analysis of the tradeoff between security and performance of WSNs in the following section.

## 7. Evaluation

Next, we evaluate our secure scheme for WSNs against timing attacks. Experiments have been conducted to investigate:

1. the effectiveness of the random padding countermeasure against timing attacks. We study how the parameter sets of the random distribution affect the mitigation effectiveness on timing attacks; and
2. the improvement of the performance and security tradeoff. From our approach, we investigate the optimal rekey interval for the WSN system under the threat of timing attacks.

### 7.1. Experiment Setup

Our experiments are developed using the OMNeT++ [33] tool based on the MiXiM [34] and the INET framework [35]. The connection between sensors and gateway nodes is enabled by the IEEE 802.15.4 standard. All tests were run under Mac OS X 10.10 on a 2.6-GHz Intel Core i5 processor with 8 GB of 1600-MHz DDR3 RAM.

Simply, a timing attack is on in which the attacker repeatedly send guesses about a secret value to the target machine, which rejects them as incorrect. However, if his or her first byte of the guess is correct, it takes a very slightly longer time to return the error. With enough measurements and proper filtering, the attacker can distinguish this difference and guess the secret correctly. The key idea of conducting a timing attack is to find the processing time differences. In order to simplify the implementation, we mimic a timing attack by recording and analyzing the gateway node response time to compare two values bit by bit.

### 7.2. Timing Attack Resilience

In this section, we investigate the timing attack resilience of WSNs with a random delay countermeasure. We evaluate the mitigation effectiveness of the countermeasure in terms of the number of additional measurements an attacker needs to make to achieve a certain percentage of successful guesses about the cluster key.

The attacker sends to the gateway node two messages with a certain bit equal to zero and one, respectively (as the two messages *g* and $g_{h}i$ in [11]), while the rest of bits are the same. After the gateway node processes each received message, random delays are padded before sending the responses. Different numbers of timing samples are taken from the attacker’s results. When the attacker can tell the time difference accurately from statistical analysis of the samples, we call it a successful guess, and we use the percentage of successful guesses to represent the mitigation influence upon timing attacks exercised by the random padding countermeasure.

**Figure 4:**
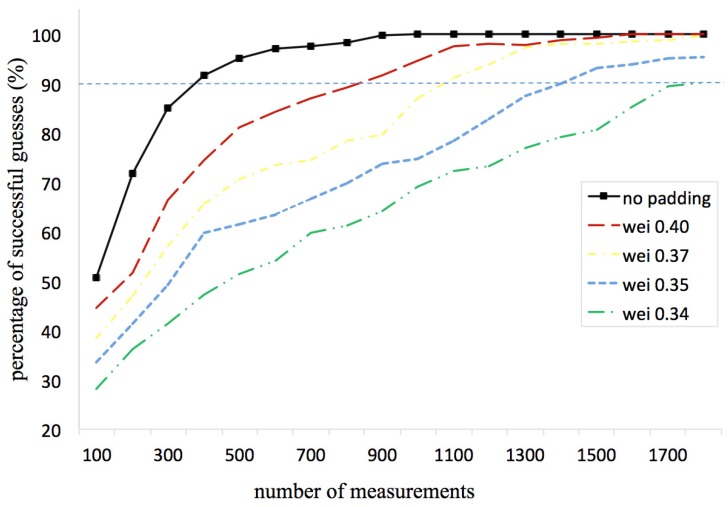
The mitigation effectiveness of different random delay paddings.

We choose Weibull distributed delay as the mitigation against timing attacks because it is easy to adjust the mean and the variance of Weibull distribution [36] by tuning the parameters, and the Weibull distribution is widely used in reliability engineering and failure analysis. We perform an experiment with different parameter sets for the Weibull distributed delays (as shown in Table 2). The experiment is conducted by changing the Weibull distribution shape parameter k∈{0.4,0.37,0.35,0.34} while keeping the scale parameter η=0.05 and making the situation when no random paddings are added as a bench mark. The result is depicted in Figure 4. One can see that when the shape parameter *k* is becoming larger, the attacking client needs more samples to guess the cluster key. As a consequence, the effectiveness of the mitigating countermeasure is becoming greater. We assume that 90 percent right guesses are adequate for a successful attack. It shows that the attacker only needs about 375 samples to get 90 percent successful guesses when no random delay is padded. While when Weibull distributed delays have the scale parameter as 0.34, 1750 samples are needed for the attacker to conduct a successful timing attack. We believe that this is due to the variance of the Weibull random delay growing with the shape parameter *k* as:

(17) $$var\left(X\right)=\eta^{2}\left[\Gamma \left(1+\frac{2}{k}\right)-{\left(\Gamma \left(1+\frac{1}{k}\right)\right)}^{2}\right]\;.$$

### 7.3. Performance and Security Tradeoff

In this section, we evaluate the different measures proposed in Section 5 and conduct the tradeoff analysis using the model analysis methods. The parameters for the model are taken from the implementation, and we use normalization. It is assumed that the attack rate on the system is $\lambda_{2}=1$. We also assume it takes an average of 0.5 time units for the gateway node to carry out a rekeying process, and the rate at which the system is brought back to the good state by the rekeying process is $\lambda_{4}=2.$ The rate at which the attacker successfully breaks the key, while failing at accessing the data or he or she just fails to conduct a successful timing attack, is assumed to be $\lambda_{6}=1.$

**Figure 5:**
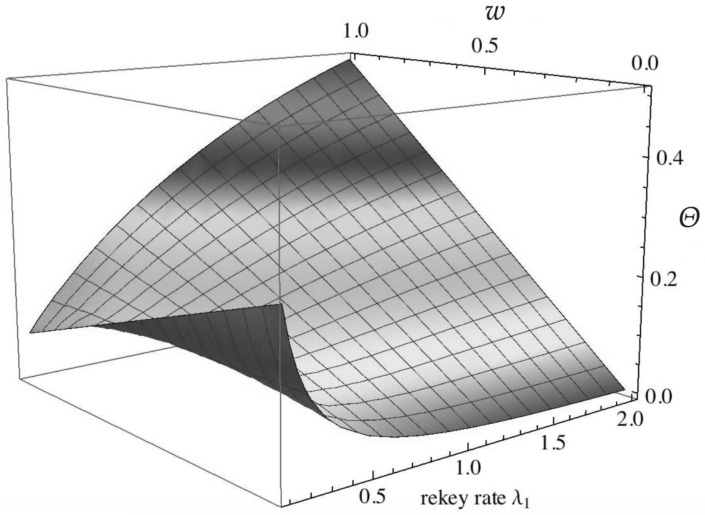
*Θ* over rekeying rate $\lambda_{1}$ and weighting parameter *w*.

Firstly, the effect of the rekeying rate $\lambda_{1}$ and the weighting parameter *w* on the system cost measure *Θ* is analyzed in Figure 5. The marginal values are investigated firstly. It is shown from the figure that when w=0, where only the costs of losing sensitive information in the compromised system are considered, the system cost decreases monotonically with the rekeying rate $\lambda_{1}$. Intuitively, in this case, when we trigger the rekeying process more often, the security cost will decrease because the WSN is more likely to be brought back to the good working state. When we put all weight on the rekeying effort (w=1), the cost increases with the rekeying rate. The light color in the middle of the figure shows the optimum rekeying rate. For the middle values of the weighting parameter *w*, the optimum rekeying rate for the lowest cost decreases when we put more weight on rekeying effort cost. For each specific rekeying rate $\lambda_{1}$, the system cost is a straight line weighting the two kinds of cost. In this figure, we get the largest rekeying effort cost and lowest security cost at rate $\lambda_{1}=2.0.$

**Figure 6:**
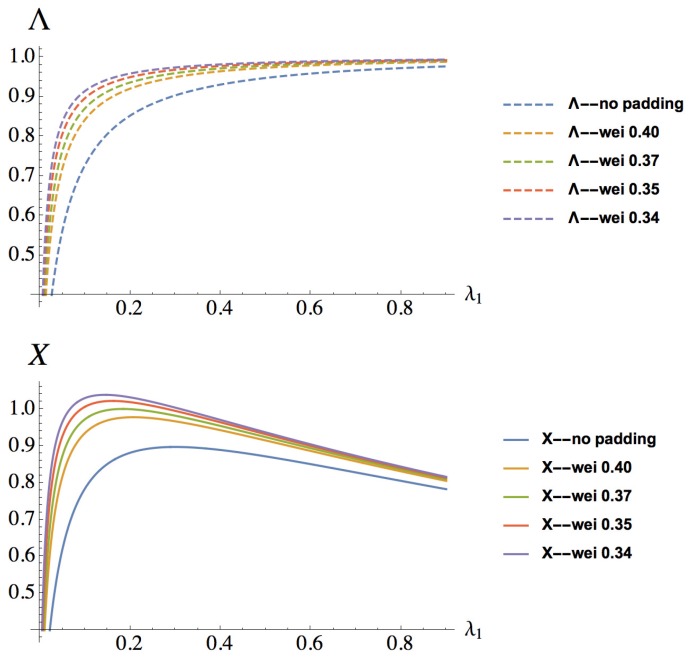
Confidentiality and throughput changing with rekeying rate $\lambda_{1}$.

Figure 6 shows the system performance and security measures, i.e., system confidentiality *Λ* (defined in Equation (2)) and throughput X (defined in Equation (3)), changing with the rekeying rate $\lambda_{1}.$ It can be seen that the confidentiality measure monotonically increases with growing rekeying rate $\lambda_{1},$ because the security improves when the system launches the rekeying process more frequently, as the system is more likely to be brought back to the good state from the timing attack state and the compromised state. While for the throughput measure, at small values of the rekeying rate, it is pretty low, because more time is spent in the compromised state when the throughput is not contributing. After obtaining the maximum throughput, the more often the gateway node triggers the rekeying act, the more often the gateway node denies sensor requests. As a result, the system throughput decreases with the rekeying rate. For a certain value of the rekeying rate $\lambda_{1}$, the performance and security of WSNs are both improved when random paddings are interposed.

**Figure 7:**
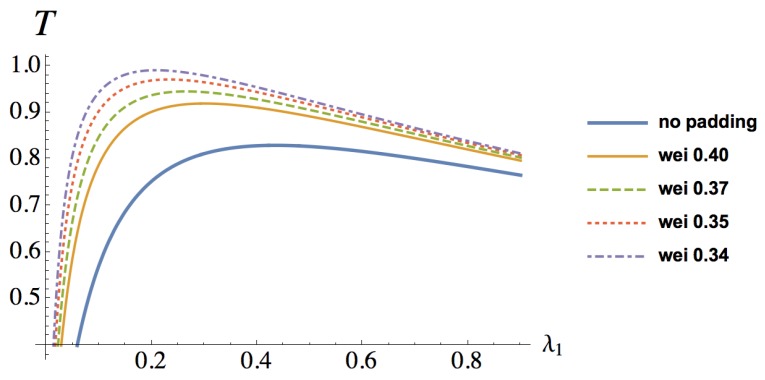
Security and performance tradeoff changing with rekeying rate $\lambda_{1}$.

At last, we present the security and performance tradeoff analysis for WSNs in Figure 7. The tradeoff measure T, defined in Equation (4), increases with the rekeying rate $\lambda_{1}$ at its low values, as the WSN security improves quickly. The rekeying rate $\lambda_{1}$ indicates how often the system launches the rekeying process. We find the optimum rekeying rate for the best security and performance tradeoff at $\lambda_{1}=0.4348,$ when no random delays are added. However, after reaching the optimum value, the tradeoff measure decreases much slower as the rekeying rate is getting larger. When the rekeying rate has a large value, the system tradeoff metric decreases because of the degrading system throughput at large rekeying rates. As Weibull random delays are padded, the tradeoff metric is greater than that with no random delay padding. This shows that the random delay countermeasure can improve the security and performance tradeoff effectively. For a specific value of the rekeying rate $\lambda_{1}$, the trade measure always improves as random delays are padded. From Table 2, it can also be seen that the optimal rekeying rate decreases when the variance of the Weibull distributed delay is growing. As a consequence, the system needs to make less effort to launch the rekeying process, which leads to less system cost. The command node can adjust the rekeying rate for the gateway node to obtain the minimum cost or maximum security performance tradeoff of the WSN.

## 8. Conclusions

A secure scheme consists of a rekeying procedure, and random delay padding is proposed for WSNs under the specific threat of timing attacks. In order to proceed to a quantitative treatment of the security-performance tradeoff, we have proposed a hybrid CTMC and queueing model for a WSN. We have shown how to formulate measures that include both security and performance attributes and that optimize the tradeoff between the two. System metrics are also proposed that take into account both the rekeying effort a system makes and the sensitive information loss. We obtained the optimal rekeying rate for the tradeoff measure.

## Figures and Tables

**Table 1:** Table of Notation.

$\pi_{i}$	≜	The steady-state probability that the continuous-time Markov process is in state i, $i\in \left\{R,C\right\}$
*Θ*	≜	System cost metric
*Λ*	≜	Confidentiality metric
X	≜	System throughput
T	≜	Tradeoff metric

**Table 2:** Optimum rekeying rate for the WSN with different parameter sets of the Weibull distribution.

Shape Para	Scale Para	Variance	n (Sample)	Optimal Rate
no padding			375	0.4348
wei 0.40	0.05	0.2725	830	0.3000
wei 0.37	0.05	0.5642	1070	0.2653
wei 0.35	0.05	0.9980	1400	0.2326
wei 0.34	0.05	1.6151	1750	0.2084
